# High-Pressure Dielectric Spectroscopic Studies of Amorphous CBD: Investigating Molecular Dynamics and Physical Stability Under Manufacturing Conditions of the Pharmaceuticals

**DOI:** 10.3390/pharmaceutics17030358

**Published:** 2025-03-11

**Authors:** Mariya Mathew, Justyna Knapik-Kowalczuk, Mateusz Dulski, Marian Paluch

**Affiliations:** 1 Institute of Physics, Faculty of Science and Technology, University of Silesia in Katowice, 75 Pułku Piechoty 1a, 41-500 Chorzów, Poland; 2 Institute of Materials Engineering, Faculty of Science and Technology, University of Silesia in Katowice, 75 Pułku Piechoty 1a, 41-500 Chorzów, Poland

**Keywords:** CBD, amorphous API, physical stability, high-pressure studies, molecular dynamics

## Abstract

**Objectives:** This study highlighted the key role played by high-pressure (HP) dielectric spectroscopic measurements of amorphous CBD to probe the molecular dynamics in order to examine the physical stability of the drug. The pharmacological properties of CBD assure that this can be a promising drug for the pharmaceutical industry. Hence, it is important to check the physical stability under elevated temperature and pressure conditions to understand the behavior of the drug under manufacturing conditions. **Methods:** This research investigated the molecular dynamics at various temperatures and pressures. We utilized the HP dielectric studies which are considered as an advanced and sensitive tool to determine both the molecular dynamics and the phase transformations. **Results:** This paper discusses the physical stability by analyzing the behavior of structural relaxation and crystallization tendencies of the amorphous drug under ambient and elevated pressure conditions. This study verified that amorphous CBD is highly physically stable at storage and elevated temperature conditions under ambient pressure. **Conclusions:** Accordingly, we examined the physical stability under elevated pressures at storage temperature, and we observed that the compression induced the crystallization of amorphous CBD. The breaking of weak hydrogen bonds present in the CBD might be the reason for this destabilization at elevated pressures. The least physical stability at high-pressure conditions was also confirmed by the broadening of the α-relaxation peak at high pressures.

## 1. Introduction

The ideal form of a pharmaceutical product is typically the solid form such as tablets, granules, and capsules [1]. Compression steps are essentially required for the processing and formulation techniques of these pharmaceutical compounds, including grinding, mixing, extrusion, fluidization, dispensing, and coating. The process of tableting involves direct compression of the pharmaceutical ingredient with additional excipients, such as lactose, starch, etc., for binding the tablet together, dispersing the active ingredient, improving the chemical and thermal stability of the active ingredient, and giving the tablet a color [2]. The pressures generated during the tableting are typically applied for a short interval of time (usually less than one second), and the pressure applied ranges between 40 and 200 MPa [3,4,5]. Moreover, it should be anticipated that the pressure has an unrivaled ability to modify the structure and properties of the pharmaceuticals [6]. All these concerns lead the manufacturing and formulation scientists to examine the effect of pressure on pharmaceuticals [7,8]. From the standpoint of processing and physical stability, crystalline drugs are excellent for the pharmaceutical industry. However, most of the crystalline drugs reveal poor aqueous solubility, impacting their low bioavailability [9,10]. One of the most frequent paths chosen to overcome this problem is the convention of the poorly soluble crystalline API (Active Pharmaceutical Ingredient) to its amorphous alternative [11,12]. The amorphous counterparts have long been recognized as a way to increase the free energy and the apparent aqueous solubility of poorly soluble pharmaceuticals [13,14]. Even though the disordered form can easily enhance the kinetic aqueous solubility of medicinal drugs, this thermodynamically unstable state reveals the tendency to recrystallize in order to achieve the low energy state [15]. Consequently, the unpredictable physical stability of amorphous APIs is the key factor that limits the extensive use of amorphous drugs [16,17]. Since the amorphous systems are marketed as solid forms, the importance of the effect of compression on the physical stability of the final product is ineluctable. Moreover, the various compression steps during the tableting and processing of solid dosage forms are associated with the various risks of crystallization [18]. Therefore, it is crucial to evaluate the effect of pressure on the prepared amorphous drugs during the manufacturing times before they are marketized.

The physical stability of an amorphous substance can be easily monitored by examining its molecular mobility [19]. Rodrigues et al. reported that Broadband Dielectric Spectroscopy (BDS) is considered a leading and non-invasive approach for determining the molecular motions of supercooled liquid and glass formers by investigating the relaxation process [20]. Many researchers have exploited BDS to investigate the temperature dependence of molecular dynamics, whereas it has been challenging to analyze the effect of pressure on molecular dynamics. Recently, our group has developed an exceptional approach to investigate the effect of elevated pressure on the physical stability of pharmaceuticals [21]. This method allows for ongoing monitoring of the amorphous pharmaceuticals’ physical stability at conditions imitating either their tableting or hot melt extrusion processes. For that purpose, the high-pressure setup for dielectric spectroscopy is employed. It has been proven that high-pressure dielectric measurements are a powerful tool to investigate the crystallization tendencies of a sample at elevated pressures. The effect of pressure on the re-crystallization of amorphous drugs is not the same for every amorphous system, as evident from our previous research. For instance, it was reported that ezetimibe and probucol were unstable at high-pressure conditions [22,23], while the behavior of indomethacin under high pressures was contradictory to the drug probucol and was observed to be stable at elevated pressures [24]. From these perspectives, we demonstrated the effect of high pressure on the CBD drug in this research. 

Pharmaceutical-grade cannabidiol has been evaluated for the treatment of seizures, and the studies revealed the medicinal benefits of CBD against these ailments [25]. In 2018, the FDA accepted the medicated drug under the brand name Epidiolex^®^ for the treatment of seizures associated with Dravet syndrome (DS) and Lennox-Gastaut syndrome (LGS) [26]. Moreover, the recent changes in regulations have sparked a renewed interest in cannabidiol for medical purposes. Numerous clinical trials are in progress to evaluate the therapeutic potential of CBD [27]. Even though the CBD drug has high pharmaceutical potential, the bioavailability and dissolution rate of crystalline CBD is very low [28] as it is categorized as a class II biopharmaceutical (poor water solubility and high permeability) [29]. Accordingly, we performed the amorphization of pure crystalline CBD to obtain the more bioavailable form [15]. To investigate the physical stability of the amorphous CBD, dielectric spectroscopy was mainly employed. Since the disordered form of CBD is relatively stable at room temperature and ambient pressure, we checked its properties and investigated its re-crystallization tendency under elevated pressure utilizing a high-pressure setup for BDS.

The present study investigated the physical stability of amorphous CBD under elevated pressures for the first time. This research reveals the effect of elevated pressure on cannabidiol at T = 298 K. This temperature seems to be important and valid because it is regarded as a long-term storage condition for pharmaceuticals [9]. Moreover, it is significant that the CBD was found to be stable at this temperature and even at high temperatures (without crystallization) under ambient pressure conditions. Our overall aim was to evaluate the compression-induced crystallization of cannabidiol, and for this purpose, the melted CBD was compressed at two different pressures and kept for a few days in order to obtain the complete crystallized sample. The pharmaceutical industries essentially need information about the influence of pressure on the pharmaceuticals in order to tablet it properly without it crystallizing and affecting the properties of the medicinal drugs. In this view, this study outthrows the effect of high pressures with the help of an excellent tool like HP BDS. Since the dielectric measurements clearly analyze the molecular dynamics at various temperatures and pressures, we demonstrate that this experimental approach becomes an efficient and competent tool to examine the molecular dynamics and, thereby, the crystallization tendencies of this cannabidiol.

## 2. Materials and Methods

### 2.1. Materials

**Figure 1:**
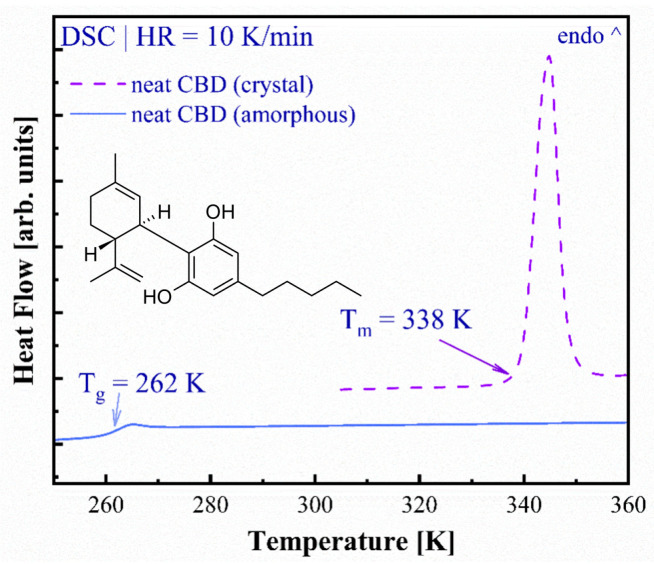
DSC thermograms of pure crystalline (violet dashed line) and amorphous (blue solid line) CBD; the inset presents the chemical structure of CBD.

The crystalline CBD isolate with a purity of 99% was purchased from Sequoya [Poland]. The CBD is chemically defined as 2-[(1R, 2R)-6-Isopropenyl-3-methylcyclohex-2-en-1-yl]-5-pentyl benzene-1,3-diol), and the chemical structure of CBD is depicted in the inset of Figure 1.

### 2.2. Experimental Methods

#### 2.2.1. Differential Scanning Calorimetry (DSC)

The thermal properties of the neat CBD were studied using a Mettler–Toledo (Zürich, Switzerland) DSC 1 STARe System. The measuring device is equipped with an HSS8 ceramic sensor having 120 thermocouples and a liquid nitrogen cooling station. The instrument is calibrated for the temperature and enthalpy using zinc and indium standards. The melting point was taken as the onset of the peak, and the glass transition temperature was the midpoint of the heat capacity increment. The samples were investigated in an aluminum crucible (40 μL). The analysis was carried out in a range from 298 K to 363 K with a heating rate of 10 K/min [23].

#### 2.2.2. Broadband Dielectric Spectroscopy (BDS)

Dielectric studies of neat CBD were carried out using a Novo-Control GMBH Montabaur, Germany dielectric spectrometer in the frequency range from 10^−1^ to 10^6^ Hz at temperatures from 173 to 331 K with steps of 2 K. The temperature was controlled by a Quattro temperature controller with temperature stability better than 0.1 K. Dielectric studies of all samples were performed immediately after fast cooling of the melt in a parallel-plate cell made of stainless steel (diameter 15 mm and a 0.1 mm gap with glass spacers). Crystallization kinetics of neat CBD were evaluated at room temperature (298 K) in order to investigate its physical stability under standard storage conditions [24].

#### 2.2.3. High-Pressure Dielectric Studies (HP-BDS)

For the pressure-dependent dielectric measurements, the specially designed high-pressure capacitor filled with the studied samples was placed in the high-pressure chamber and compressed using silicone oil. During the measurement, the samples were only in contact with stainless steel and Teflon. The Nova Swiss tensometric pressure meter with a resolution of 0.1 MPa was used to measure the pressure. The temperature was controlled within 0.1 K by means of a liquid flow provided by a Weiss fridge and Julabo thermal bath [23].

The experimental procedures adopted for determining the isotherms and crystallization kinetics were different. For evaluating the four isotherms, the temperature was set up and maintained for nearly 90 min in order to ensure that the sample got the exact temperature as applied via the thermostat. Thereafter, the sample placed inside the oil chamber was compressed beyond the glass transition region in order to avoid crystallization. The sample was allowed to wait for 15 min after the application of every single pressure condition before starting the measurement.

For examining the crystallization kinetics, the sample placed inside the oil chamber was subjected to the required pressure condition (20 MPa, 40 MPa). The measurement was started 30 min after the application of pressure. In both cases, the teflon spacer was used between the capacitors, and the sample inside the capacitors was secured entirely from the disturbances of oil by proper teflon taping.

#### 2.2.4. Fourier Transform Infrared Spectroscopy (FTIR)

The FTIR measurements of crystalline and melted CBD were performed using a Cary 640 FTIR spectrometer (Agilent Technologies, Stevens Creek Blvd, Santa Clara, CA, USA) equipped with a standard source and a DTGS Peltier-cooled detector. The spectra were collected in the 400–4000 cm^−1^ range using a GladiATR diamond accessory. Each spectrum was recorded by accumulating 16 scans with a spectral resolution of 4 cm^−1^. Infrared data were post-processed using Win-IR Pro (v2.96) with baseline correction, water, and carbon dioxide removal, as well as ATR correction, considering the refractive index of CBD (*n* = 1.5404).

## 3. Results and Discussion

### 3.1. Thermal Properties of Crystalline and Amorphous CBD

The differential scanning calorimetry technique was exploited to measure the thermal properties of both crystalline and amorphous CBD. Figure 1 shows the DSC thermograms obtained during sample heating with a rate of 10 K/min. The first heating curve, represented as a violet dashed line, presents the thermogram of the crystalline (as obtained) form of CBD. Herein, one can notice the sharp endothermal peak corresponding to the melting of this API, with the onset registered at 338 K. It was possible to achieve the amorphous form of CBD by the rapid (with the cooling rate equal to 20 K/min) quenching of molten CBD. The second heating curve, presented in a blue solid line, shows the thermogram of amorphous CBD. The glass transition temperature (T_g_) of this material has been found to be 262 K based on the midpoint of the registered step-like event. It is worth noting that the amorphous CBD did not recrystallize during the second heating at this heating rate of 10 K/min, and it is evident from the absence of an exothermic peak in the mentioned curve. The observed melting point was found to be nearly similar to the T_m_ (340–341 K) reported by previous studies [30,31].

#### Molecular Features of Crystalline and Molten CDB

Infrared spectroscopy was employed to investigate the influence of intra- and intermolecular interactions on the molecular network arrangement of cannabidiol (CBD). According to crystallographic data, CBD crystallizes in the monoclinic space group P21. Within the crystal unit, CBD molecules adopt an H-bond-stabilized configuration, wherein two independent molecules exist in the asymmetric unit with distinct orientations of the unsaturated alkyl side chain [32].

In conformer A, the n-pentyl group aligns linearly with the aromatic ring, whereas in conformer B, it is rotated. The terpene and phenolic rings of CBD are nearly perpendicular to each other. The presence of hydroxyl groups and aromatic moieties facilitates intermolecular interactions between specific molecular units, contributing to the supramolecular arrangement of CBD molecules.

Two distinct crystal structures have been described: in crystal state I, weak H-bond interactions occur between conformers A and B, characterized by an O(1)-H···O(2) bond distance of 2.859 Å [33]. In contrast, crystalline state II results from a slight rotation of one hydroxyl group, leading to a stronger H-bond interaction between conformers A and B, with an O(1)-H···O(2) bond distance of 1.985 Å [32]. Furthermore, supramolecular arrangements contribute to the stabilization of the CBD molecular network via slightly stronger C-H···O interactions between methyl and hydroxyl groups. Additionally, dihydrogen interactions enhance the stability of crystal state I, while C-H···π interactions between conformers A and B, or two B conformers, provide further stabilization in a state I or II, depending on aromatic hydrogen positioning [34].

**Figure 2:**
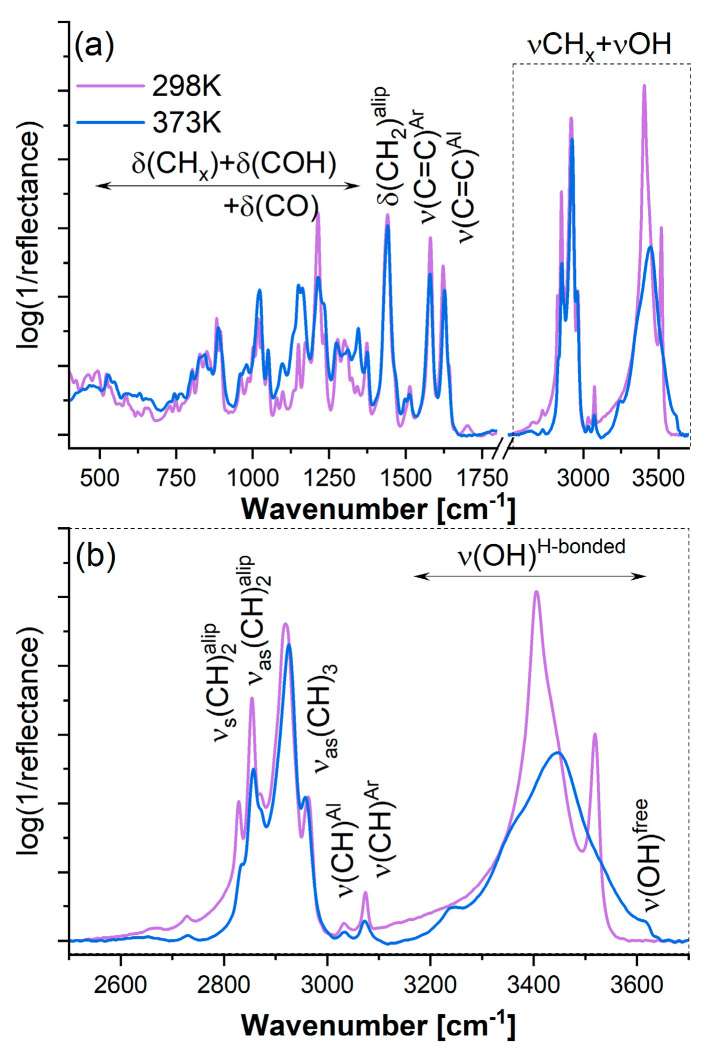
Infrared spectra of pure crystalline (violet solid line) and amorphous (blue solid line) CBD summarized in the 400–3700 cm^−1^ range (**a**) as well as 2550–3700 cm^−1^ (**b**).

Infrared spectroscopy observations well support these structural characteristics. The fingerprint region reveals multiple characteristic bands: C=C stretching of alicyclic moieties (1620 cm^−1^), C=C stretching of the aromatic ring (1580 cm^−1^), -CH_2_- bending of the aliphatic chain (1440 cm^−1^), deformation vibrations of O-H and C-O stretching (1410–1310 cm^−1^), and CH_x_ deformation modes below 1000 cm^−1^, corresponding to the out-of-plane deformation of the O-H group (720–600 cm^−1^) (Figure 2a).

The most significant interpretation of the hydrogen bonding scheme is observed in the high-wavenumber region, where symmetric and asymmetric stretching modes of CH_x_ appear between 2800 and 3100 cm^−1^. Two clearly visible and intense bands at approximately 3518 and 3405 cm^−1^ originate from the stretching vibration of the O–H group [35,36]. Their positions are highly influenced by intermolecular interactions resulting from the supramolecular configuration (Figure 2). The asymmetry in the lower hydroxyl-related band further supports the formation of a supramolecular system. Notably, the high-wavenumber band originates from the vibration of unassociated phenolic hydroxyl groups, whose position is determined by C-H···O interactions. Meanwhile, the other two bands arise from highly localized O-H···π interactions and stronger intermolecular hydrogen bonding between two conformers, with O-H···O interactions and a donor-acceptor bond distance of 1.985 Å, characteristic of crystal state II.

The hydrogen bond network undergoes modifications upon sample melting. As a result of amorphization, the infrared spectrum of the amorphous sample exhibits numerous lower-intensity and significantly broadened bands in the fingerprint region, corresponding to various molecular configurations. However, similar to crystalline observations, multiple bands associated with CHx symmetric vibrations (2800–3100 cm^−1^) were detected. Additionally, one clearly visible band of lower intensity and increased full width at half maximum (FWHM) indicates a broad distribution of hydrogen bonds with varying donor-acceptor bond lengths.

During amorphization, the supramolecular structure characteristic of the crystalline state is disrupted, giving rise to new molecular interactions. These include weak, medium, and strong hydrogen bonds, as evidenced by bands at 3447, 3354, and 3239 cm^−1^. Furthermore, the formation of novel molecular arrangements results in non-interacting molecules or molecules with free phenolic hydroxyl groups that do not engage in intermolecular interactions (Figure 2).

### 3.2. Molecular Dynamic Studies of Pure Amorphous CBD to Examine the Physical Stability

**Figure 3:**
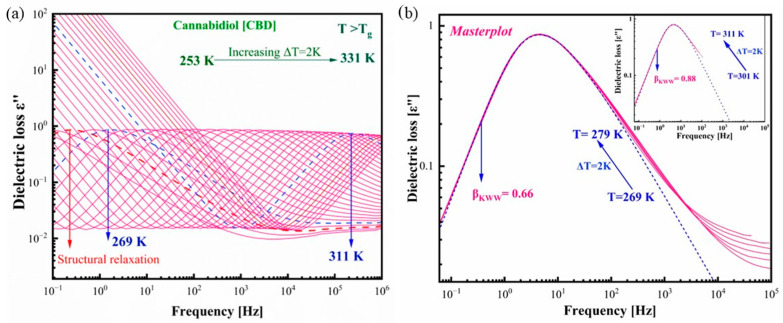
Dielectric spectra of CBD obtained upon heating. (**a**) Spectra obtained above the glass transition temperature of CBD. (**b**) Master plot elaborated by superimposing the dielectric spectrums from T = 269 K to T = 311 K (above the T_g_). The dashed blue line presents the KWW fit.

The isobaric dielectric studies were conducted to probe the molecular dynamics of amorphous CBD in a wide frequency and temperature range. The frequency explored in the study varied from 10^−1^ to 10^6^ Hz and the temperature from 173 K to 258 K with a step of 5 K as well as from 259 to 331 in 2 K steps. Figure 3a presents the dielectric spectra obtained above the glass transition temperature of CBD. The spectra clearly reveal the prominent α-relaxation of the CBD drug, which is attributed to the liquid structural reorganization of the CBD molecules. The α (structural) relaxation peaks shift towards higher frequencies with increasing temperature. The shifting of structural relaxation towards low frequencies with lowering temperature is due to the increase in the α-relaxation time, which is considered the characteristic element of structural relaxation [37]. There was no pronounced secondary relaxation in the above and below the glass transition temperatures. Thus, it is obvious that the only molecular dynamics observed in the CBD drug are from the structural relaxation process.

The temperature dependence of the shape of the structural α-relaxation can be easily visualized by plotting the master plot in which the spectra measured at different temperatures are horizontally shifted and superimposed on the reference spectrum. Figure 2b shows the master plot in which the spectra from 269 K to 279 K are taken for analysis. It is clear from Figure 3b that the temperature affected the shape of α-relaxation, and the spectra are slightly broadened at lower temperatures (near the glass transition). The shape of the α loss peak can be characterized by a parameter known as the stretching parameter (β_KWW_). The value of β_KWW_ was determined by fitting the α-relaxation spectra by Fourier transform of the Kohlrausch–Williams–Watts stretched exponential function (KWW) [38]. Shamblin et al. reported that the stretching parameter β_KWW_ has a key role in evaluating the physical stability of amorphous pharmaceuticals [39]. According to their interpretation, the higher the β_KWW_ value, the higher the physical stability of the amorphous drugs should be against crystallization. It is mostly observed that the shape of structural relaxation peaks was unchanged with increasing temperature for the amorphous systems like nonivamide β_KWW_ = 0.79 [40], ketoprofen β_KWW_ = 0.71 [41], indapamide β_KWW_ = 0.72 [42], and probucol β_KWW_ = 0.6 [23]. On the contrary, the shape of α-relaxation is sensitive to temperature for the CBD drug. The β_KWW_ f CBD for spectra at lower temperatures (269 K to 279 K) was found to be β_KWW_ = 0.66. The inset in Figure 3b presents the master plot at higher temperatures (301 K to 311 K), and the β_KWW_ value for the spectra was observed to be β_KWW_ = 0.88. Taking into account Shamblin’s postulate, one could conclude that the narrow spectrum of CBD indicates the higher physical stability of this amorphous drug. The performed isothermal crystallization kinetics under ambient conditions indeed proved the resistance of CBD against crystallization at high temperatures. The β_KWW_ values of the above-mentioned amorphous drugs are nearly the same. Consequently, the physical stability is expected to be similar for all the mentioned amorphous systems. It is worth noting that the amorphous indapamide is stable at a wide range of temperatures and no crystallization has been observed even one year after amorphization (storage conditions: T = 293 K, humidity 10%). Our previous dielectric studies of probucol revealed that it was as good of a glass former as that of CBD under ambient pressure conditions [23]. Moreover, Sailaja et al. reported that ketoprofen was a non-crystallizing compound from the calorimetric (DSC) experiments [41]. From the stretching parameter values of probucol, indapamide, and ketoprofen, one could conclude that CBD will also be a good glass former at storage temperature as well as even at higher temperature conditions, which is also reflected in the narrowing of the dielectric loss spectra at higher temperatures.

**Figure 4:**
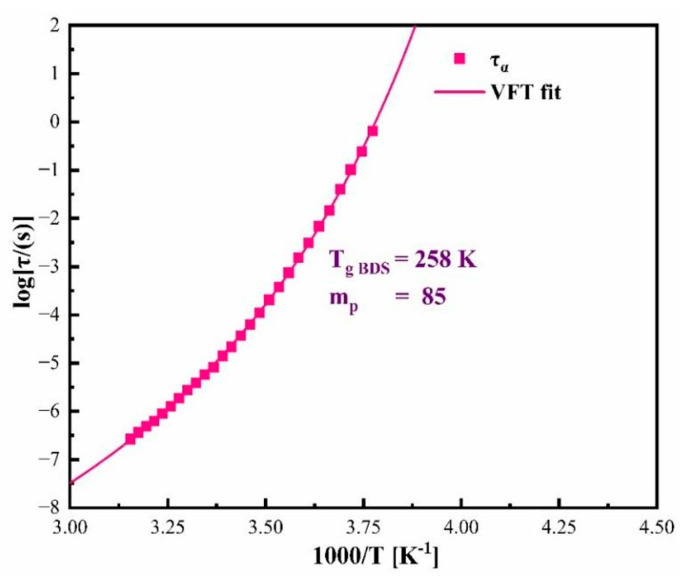
The temperature dependence of τ_α_ in the supercooled liquid region. The pink solid line represents the VFT fit to the experimentally determined dependence (pink squares).

The measured dielectric loss spectra were capable of evaluating the temperature dependence on the α-relaxation time (*τ_α_*), which is depicted in Figure 4. The α-relaxations were fitted using the Havrilak–Negami (HN) function in order to determine τ_α_. The empirical HN function can be defined by Equation (1) [43]:

(1) $$\varepsilon_{HN\;}^{*}\;\left(\omega \right)=\varepsilon^{\prime }\left(\omega \right)-i\left(\omega \right)=\varepsilon_{\infty }+\frac{∆\varepsilon }{[{1+{\left(i\omega \tau_{HN}\right)}^{a}]}^{b}}$$

where ε′ and ε″ are real and imaginary parts of complex dielectric permittivity, ε_∞_ is high frequency limit permittivity, Δε is dielectric strength, ω is equal to 2πf, τ_HN_ is the HN relaxation time, and *a* and *b* represent symmetric and asymmetric broadening of the relaxation peak. The obtained fitting parameters can then be used to calculate τ_α_ using Formula (2):

(2) $$\tau_{\alpha }=\tau_{HN}\;{\left[sin\;\left(\frac{\pi a}{2+2b}\right)\right]}^{\frac{-1}{a}}\;{\left[sin\;\left(\frac{\pi ab}{2+2b}\right)\right]}^{\frac{-1}{a}}$$

The experimentally determined α-relaxation time of CBD at supercooled region can be well-described using the Vogel–Fulcher–Tammann (VFT) equation, defined as follows:

(3) $$\tau_{\alpha \;}\left(T\right)=\tau_{\infty }\;exp\left(\frac{B}{T-T_{0}}\right)$$

where τ_∞_, T_0_, and B are the fitting parameters [44,45,46] and the corresponding fitting parameters for CBD equal to τ_∞_ = −13.29 ± 0.14, T_0_ = 211.21 ± 1.07, and B = 7.73 ± 0.27. The glass transition temperature of the investigated pharmaceutical can be obtained from the data by calculating the temperature corresponding to τ_α_ = 100 s (T_g_ = T (τ_α_ = 100 s)). The glass transition temperature calculated from VFT is equal to 258 K, and this value is in good agreement with the glass transition temperature obtained from DSC experiments conducted at a heating rate of 10 K/min, equal to 262 K.

Another parameter often used to characterize the physical stability of glass-forming liquids is the fragility [47]. The fragility parameter (*m_p_*), commonly known as the steepness index, is a measure of the degree of deviation of the temperature dependence of structural relaxation times from the Arrhenius law at the glass transition temperature [40] and is defined as follows [48]:

(4) $$m_{p\;}={\;\left[\frac{d{log}_{10}\;\tau_{\alpha }}{d\left(\frac{T_{g}}{T}\right)}\right]}_{T=Tg}$$

According to Angell’s interpretation, this parameter can be used to identify whether the prepared amorphous material is fragile, intermediate fragile, or strong when *m_p_* ≥ 100, 30 < *m_p_* < 100, or *m_p_* ≤ 30, respectively. More importantly, it has been suggested that strong glass formers become physically more stable than fragile ones. The fragility parameter of the investigated amorphous CBD is equal to *m_p_* = 85. Consequently, the amorphous CBD can be classified as an intermediate glass former according to Angell’s classification, and it is expected to have intermediate stability. In contrast to this assignation, CBD was found to be substantially stable against crystallization. This contradictory observation can be rationalized by recalling the experimentally established correlation between the fragility parameter and β_KWW,_ which is captured by Equation (5) [48]:

(5) $$m_{p}=m_{0}-s\beta_{KWW}$$

where *m*_0_ and *s* are constants with values *m*_0_ = 250 and *s* = 320. By substituting this value and *β_KWW_* of CBD (*β_KWW_* = 0.66) into Equation (5), the value of fragility of CBD is found to be *m_p_* = 38.8. This value suggests that the prepared amorphous CBD can be classified as a strong glass former. However, the fragility value obtained from Equation (4) is equal to *m_p_* = 85. This deviation in the fragility value shows that the amorphous CBD is not satisfying this Angell’s correlation. There are several examples demonstrating that fragility is not always a good predictor of physical stability. For instance, the fragility parameter of amorphous ketoprofen was *m_p_* = 86.57. However, the drug was observed to be non-crystalline in DSC experiments by Sailaja et al. [41]. The fragility of extensively stable amorphous indapamide was recorded as *m_p_* = 76, and according to the classification by Angell, it should fall into the former fragile glass category. Although the fragility value is explicit that the amorphous indapamide is fragile, the dielectric experiments by Wojnarowska et al. proved its stability against crystallization, and it was observed that this drug was purely amorphous during the entire storage period of one year [42,49]. Hence, one could conclude that the fragility parameter of CBD cannot be considered as a main factor for determining the physical stability against crystallization.

### 3.3. Effect of Elevated Pressure on the Re-Crystallization Tendency of Pure Amorphous CBD

As explained in the introduction, the amorphous forms of drugs are appropriate candidates for improving bioavailability. However, their main drawback is related to their limited physical stability. Hence, it is important to discuss the physical stability of drugs at the storage conditions of pharmaceuticals. It was pointed out that the amorphous CBD does not recrystallize at storage, as well as elevated temperature conditions under ambient pressure. Since our study is focused on the amorphous CBD for pharmaceutical applications, it is crucial to consider the effect of elevated pressures on the prepared amorphous CBD. The effect of pressure on amorphous drugs is important because of the compressions applied during the formulation processes.

**Figure 5:**
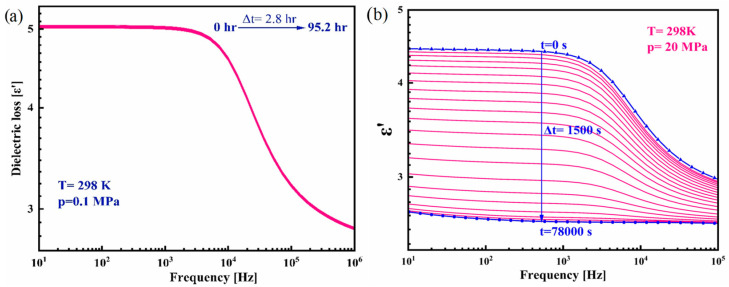
The real part of dielectric spectra of amorphous CBD recorded during an isothermal crystallization at T = 298 K under (**a**) p = 0.1 MPa and (**b**) p = 20 MPa.

The isothermal cold crystallization of CBD was investigated by HP-BDS, which is a very sensitive method to examine the changes in molecular dynamics and the crystallization tendencies associated with the changes in the applied pressure. To examine the physical stability of amorphous CBD at elevated pressure, the isothermal crystallization kinetics at T = 298 K and p = 0.1, 20, and 40 MPa were carried out. Figure 4 shows the real part of the dielectric spectra measured as a function of time under ambient pressure (Figure 5a) and p = 20 MPa (Figure 5b). Due to the fact that the storage condition for pharmaceuticals is 298 K, we performed the isothermal crystallization at the same temperature (T = 298 K). In order to evaluate the influence of pressure on the crystallization, the experiments were carried out in two different elevated pressure conditions (20 and 40 MPa) as well as at ambient pressure as a reference point. It is understood from the figure that there is no re-crystallization under ambient conditions. However, a small pressure of p = 20 MPa caused CBD to be crystallized entirely within 20 h. Such a noticeable drop in the dielectric strength of the structural relaxation Δε_α,_ as observed in Figure 5b is typical for the crystallization process, which is attributed to the decrease in the amorphous fraction of CBD [50]. The progress of devitrification of amorphous CBD was examined in terms of the normalized real permittivity *ε*′*_N_*, given as follows [51]:

(6) $${\varepsilon \prime }_{N}\;(t)=\frac{\varepsilon \prime \;\left(0\right)-\varepsilon \prime \;\left(t\right)}{\varepsilon \prime \;\left(0\right)-\varepsilon \prime \;\left(\infty \right)}\;$$

where *ε*′(0) and *ε*′(*∞*) are the values of static dielectric permittivity at the initial and ultimate stages of the re-crystallization process, while *ε*′(*t*) is the value at time *t*. Figure 5a,b clearly compares the behavior of the physical stability of amorphous CBD under ambient and elevated pressure conditions.

**Figure 6:**
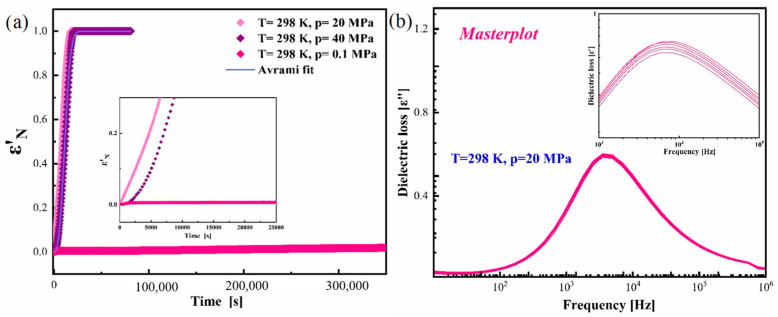
(**a**) Normalized dielectric constant (ε′_N_) as a function of time from the physical stability test at T = 298 K and p = 0.1, 20 and 40 MPa. (**b**) Master plot of the dielectric spectra collected at T = 298 K and p = 20 MPa.

In order to analyze the progress of crystallization with time, the data from time-dependent isothermal dielectric studies were normalized using Equation (6) and are shown in Figure 5a. From this figure, it is clear that elevated pressure destabilizes the amorphous CBD system, whereas the amorphous CBD is very stable at ambient pressure and the same T conditions. The reason for the faster re-crystallization of amorphous CBD at elevated pressure might be due to the breaking of H bonds, which leads to the crystallization. The Avrami model is most frequently used to define the crystallization kinetics under isothermal conditions. We describe the time dependences of ε′_N_ for CBD by using the following modified Avrami equation [50]:

(7) $${\varepsilon \prime }_{N\;}\left(t\right)=1-exp{\left(-Kt\right)}^{n}$$

where *K = k^n^* is the crystallization rate constant, and *n* is the Avrami parameter that is directly related to the nucleation dimensionality. Usually, parameter n falls in the range of 2−3. From Equation (7), an Avrami plot was constructed to obtain the time dependence on the crystallization of amorphous CBD. The value of fitted parameters was equal to *k* = 7.65 × 10^−5^ and *n* = 2.7. The inset of Figure 6a shows that at higher pressure (p = 40 MPa), the re-crystallization progress was slowed down compared to that at p = 20 MPa. Figure 6b displays the master plot of the spectrum from the dielectric spectra measured at p = 20 MPa by superimposing the spectrum at different time intervals on the reference spectrum. The inset in Figure 5b clearly demonstrates that although there is a drop in the intensity of dielectric loss, there is no change in the shape of the structural relaxation even after 50% of the sample gets crystallized.

### 3.4. Effect of Elevated Pressure on the Structural Relaxation of Pure Amorphous CBD at Various Isothermal Conditions

**Figure 7:**
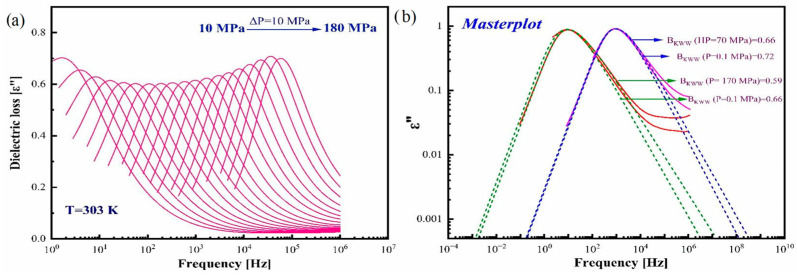
(**a**) Dielectric loss spectra of CBD obtained at various pressures at isotherm T = 303 K. (**b**) Master plot obtained by superimposing the dielectric spectra of ambient pressure and elevated pressure at isochronal conditions (*f* = 10^3^ Hz and 10^1^ Hz).

To obtain a deeper understanding of the impact of elevated pressure on the molecular dynamics of amorphous CBD, the dielectric spectra of CBS’s α-relaxations at various pressures under different isothermal conditions (T = 279, 286, 293, and 303 K) were investigated during sample compression with a step of 10 MPa. The spectra measured at isotherm T = 303 K are shown in Figure 7a as an example. Due to the pronounced tendency of amorphous CBD to crystallize at elevated pressure, the four isotherms were achieved by direct, fast compression to the glass transition pressure followed by decompression at isothermal conditions. The α-relaxation peak shifts toward higher frequencies during decompression. The effect of pressure on the shape of the dielectric spectra was inspected by comparing the two spectra, one at ambient pressure and the other at elevated pressure at the same relaxation time (τ_α_), which are depicted in Figure 7b. In addition, each spectrum was fitted to the KWW function. It can be clearly seen that the β_KWW_ value decreases with pressure, indicating that the spectrum gets broadened at high-pressure conditions. This broadening of the spectrum is most likely related to the reorganization of weak hydrogen bonds, which facilitates the molecular ordering of CBD molecules due to the applied pressure and the formation of a supramolecular structure distinct from that of the neat CBD structure, as suggested by diffraction data. According to the literature reports, this alternative supramolecular arrangement may induce the crystallization of CBD in crystal state I. It is worth noting that this pattern of behavior nicely agrees with the Shamblin correlation. Thus, the broadening of the spectrum with the increase in pressure reflects the higher tendency of amorphous CBD to crystallize. The weakness of the hydrogen bonds can also be understood from the dT_g_/dp value, which is discussed later. 

The measured dielectric loss spectra were capable of evaluating the pressure dependence on the α-relaxation process at different isothermal conditions (T = 279, 286, 293, and 303 K). The α-relaxations at various pressures under various isothermal conditions were fitted using the HN function. For most glass formers, the continuous increase of the α-relaxation time during compression can be well parameterized by the pressure counterpart of the VFT law (p-VFT), which is defined as follows [52]:

(8) $$\tau =\tau_{0\;}exp\frac{D_{p\;}p}{p_{0}-p}\;$$

where *τ*_0_ is the relaxation time at ambient pressure, *p*_0_ is the pressure of ideal gas, and *D_p_* is the parameter that relies on the pressure fragility of the material. The fitting parameters (*τ*_0_, *p*_0_, and *D_p_*), activation volume from the fitted curve (Δ*V^#^*), and pressure corresponding to glass transition (*p_g_*) for the four isothermal conditions are tabulated in Table 1.

**Figure 8:**
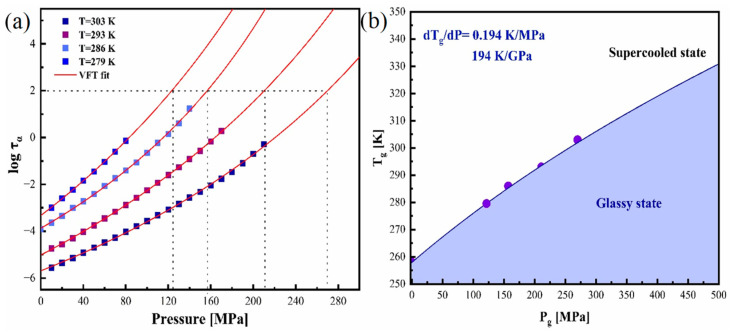
(**a**) The pressure dependence of τ_α_ of supercooled CBD explained using p-VFT equation. (**b**) Diagram representing T_g_-p_g_ dependence of CBD.

From the p-VFT fit, it was possible to calculate the pressure corresponding to glass transition (*p_g_* = *p* (*τ_∞_* = 100 s)) at various isotherms. The p-VFT fit of four isotherms at various pressures is shown in Figure 8a.

The p-VFT equation was used to evaluate the pressure corresponding to glass transition and was recorded as p = 270, 211, 157, and 125 MPa for isothermal conditions T = 303, 293, 28, and 279 K, respectively. The values clearly show that the pressure at the glass transition region increases with the temperature. The variation in the glass transition temperature with pressure is displayed in Figure 8b. The characteristic feature of T_g_(p) dependence is the nonlinear increase in T_g_ with pressure. The T_g_-p_g_ dependence obtained was fitted by the Avramov equation (Equation (9)) [43]:

(9) $$T_{g}(p)=T_{g}(p_{0}){\left(1+\frac{p}{\pi }\right)}^{a}$$

The T_g_(p_0_) and T_g_(p) are glass transition temperatures at ambient pressure and specific pressures, and π and *a* are constants. The values of the fitted parameters were T_g_ = 258, π = 424, and *a* = 0.319, respectively. The fitted curve was exploited to deduce the value of the pressure coefficient from their initial slope, (dT_g_/dp) p → 0, which is equal to 194 K/GPa. For H-bonded systems like glycerol, the extensive hydrogen-bonding network hinders the material from responding to the compression, which is reflected in the low dT_g_/dp value equal to 40 K/GPa. For a typical Van der Waals system, the dT_g_/dp value was found to be significantly high, greater than 150 K/GPa [26]. The glass transition temperature of CBD increased nonlinearly with the applied pressure with a dT_g_/dp = 194 K/GPa, implying the relatively high sensitivity of the structural relaxation dynamics to compression. The dT_g_/dp value of CBD is much higher than that of glycerol. Considering the structure and dT_g_/dp value of CBD, we can conclude that the hydrogen bond present in the CBD is weak. Since the hydrogen bond is weak, elevated pressure might reorganize it, thereby modifying the physical stability, which can be noticed from the broadening of structural relaxation peaks at high pressures. This broadening can be easily understood from the variations in the β_KWW_ values at low and elevated pressures (β_KWW_ = 0.66 for p = 70 MPa; β_KWW_ = 0.59 for p = 170 MPa). The dT_g_/dp value of the S-enantiomer of ketoprofen was found to be almost the same as that of CBD, dT_g_/dp = 193 K/GPa [53]. The structure of ketoprofen also possesses the hydroxyl group, and it was observed that upon increasing the pressure, crystallization is induced for the S-enantiomer [54]. The behavior of ketoprofen (S-enantiomer) can be regarded as similar to that of CBD as it also behaves more like a Van der Waals system. From these data, one can suppose that the glass dynamics of weak hydrogen bonding systems are quite sensitive to pressure.

### 3.5. Determination of Activation Volume of Amorphous CBD

**Figure 9:**
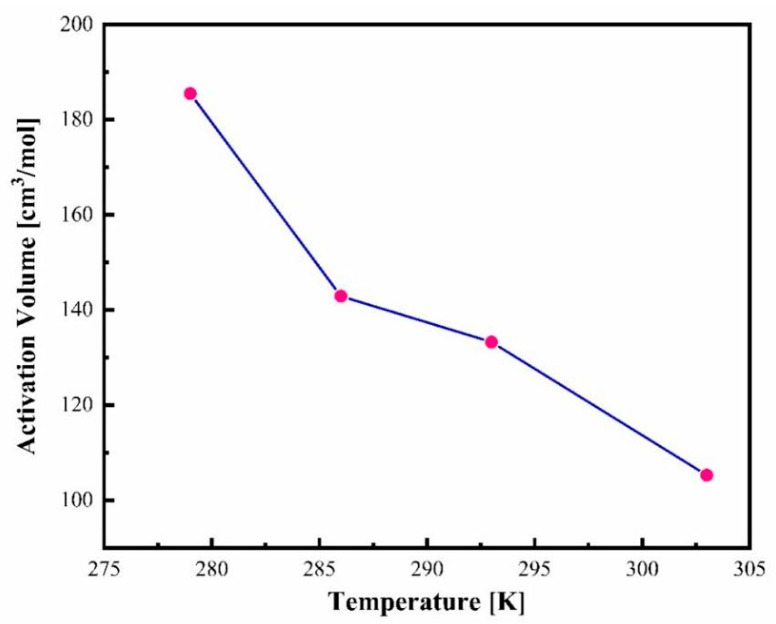
The temperature dependence of activation volume of CBD.

Activation volume is regarded as a crucial parameter that characterizes the sensitivity of α-relaxation to pressure [55]. From the VFT fitting curve of isothermal pressure dependence on structural relaxation (Figure 7a), we were able to determine the activation volume using Equation (10) [52] at various temperatures under ambient pressure.

(10) $$∆V=2.303\;RT\;{\left(\frac{\partial log\tau_{\alpha }\;}{\partial P}\right)}_{T}$$

where *R* is the universal gas constant, T is temperature, and *τ_α_* is the α-relaxation time. The effect of temperature on activation volume at the limit of ambient pressure is depicted in Figure 9. From this figure, it is clear that the value of Δ*V^#^* increases with the lowering of temperature. This increase in activation volume with decreasing temperature is attributed to the increase in the heterogeneity of the dynamics of the amorphous CBD drug [43].

## 4. Conclusions

This paper discussed the physical stability of the prepared amorphous CBD using dielectric experiments. The DSC technique initially studied the thermodynamic behavior, and relaxation studies were investigated by BDS. It was interesting to note that the amorphous CBD was substantially stable against crystallization under ambient pressure, even at elevated temperatures. The dielectric loss spectra of amorphous CBD clearly showed the narrowing of the structural relaxation peak at higher temperatures, also verifying the enhanced physical stability at higher temperatures. Since the study aimed at developing pharmaceutical formulations, HP dielectric studies were conducted to evaluate the impact of pressure on molecular mobility. Consequently, the amorphous CBD system was subjected to various high-pressure conditions at p = 20 and 40 MPa at T = 298 K. We explored this temperature for the crystallization kinetics due to the fact that it is regarded as the storage condition for pharmaceuticals. From the compression studies, we inferred that the amorphous CBD becomes destabilized even at the small pressure p = 20 MPa. However, it is exciting to note that the crystallization was slower at p = 40 MPa compared to that at p = 20 MPa. The impact of pressure on the molecular dynamics was broadly analyzed by performing different isotherms (T = 303, 293, 286, and 279 K), and from the VFT fitting curve and T_g_-p_g_ dependency, we determined the dT_g_/dp = 194 K/GPa. This value suggests that the hydrogen bond present in the CBD is weaker because the dT_g_/dp value is closer to that of the Van der Waals system than that of the H-bonded system. This information reveals that during compression, the H bonds might be reorganized, and this disturbs the physical stability, which leads to crystallization. The lower physical stability at higher pressures is also reflected in the broadening of the dielectric loss spectrum (structural relaxation peak) at elevated compression stages.

## Figures and Tables

**Table 1:** The comparison of p-VFT parameters together with Δ*V^#^* and *p_g_* of amorphous CBD measured at different isotherms.

Isotherms	τ_0_	p_0_	D_p_	ΔV^#^ (cm^3^/mol)	P_g_ (MPa)
279 K	−3.33 ± 0.02	625 ± 80	49.9 ± 7.5	186	125
286 K	−387 ± 0.03	513 ± 38	30.8 ± 3.1	143	157
293 K	−5.01 ± 0.02	730 ± 33	39.9 ± 2.4	133	211
303 K	−5.69 ± 0.03	741 ± 49	31.0 ± 2.8	105	270

## Data Availability

Data is contained within the article.
